# miR-155 suppresses angiotensin II type 1 receptor synthesis during placental morphogenesis

**DOI:** 10.1038/s41420-025-02892-0

**Published:** 2025-12-24

**Authors:** Anya L. Arthurs, Eugenie R. Lumbers, Lachlan Schofield, Celine Lees, Peck Y. Chin, Alison S. Care, Sarah A. Robertson, John E. Schjenken, Kirsty G. Pringle

**Affiliations:** 1 Flinders University, College of Medicine and Public Health, Flinders Health and Medical Research Institute, Adelaide, SA Australia; 2 School of Biomedical Sciences and Pharmacy, College of Health, Medicine and Wellbeing, University of Newcastle, Callaghan, NSW Australia; 3 Women’s Health Research Program, Hunter Medical Research Institute, New Lambton Heights, NSW Australia; 4 Robinson Research Institute and School of Biomedicine, The University of Adelaide, Adelaide, SA Australia; 5 Centre for Reproductive Science, School of Environmental and Life Sciences, College of Engineering, Science and Environment, University of Newcastle, Callaghan, NSW Australia; 6 Infertility and Reproduction Research Program, Hunter Medical Research Institute, New Lambton Heights, NSW Australia

**Keywords:** Morphogenesis, miRNAs, Differentiation

## Abstract

Several microRNAs play vital roles in placental development. *miR-155* has been implicated in placental development and can directly interact with a variety of targets, including angiotensin type II receptor 1 (AT_1_R) (*Agtr1*) mRNA. The AT_1_R is pro-proliferative and promotes early placental development. We therefore tested the hypothesis that *miR-155* downregulates *Agtr1* mRNA expression and impairs placental development. Placentae and fetuses from wild-type C57Bl/6 mice (*miR-155*^+/+^, control) and C57Bl/6 mice with a null mutation in *miR-155* (*miR155*^-/-^) were mated with males of the same genotype and analyzed on gestational day 18.5, when placental morphology and *miR-155* and *AGTR1* expression were assessed. Additionally, HTR8/SVneo cells were cultured with a *miR-155* mimic to determine the effects on trophoblast proliferation, migration and invasion. *miR-155*^-/-^ dams produced significantly heavier pups with unchanged placental weights and fetal-to-placental weight ratios. Placentae from miR-155^-/-^ dams had significantly larger labyrinth zones and labyrinth-to-placental area ratios than controls, with altered stereological parameters. Placental *Agtr1* mRNA and AGTR1 protein levels were significantly increased in *miR-155*^-/-^ dams. Finally, in vitro treatment in human HTR-8/SVneo cells with the *miR-155* mimic increased *miR-155* expression, decreased *AGTR1* mRNA levels and decreased the rates of trophoblast cell proliferation, migration and invasion. Thus, *miR-155* is demonstrated to attenuate placental development in mice. We propose that this is at least partly due to its effects on the AT_1_R.

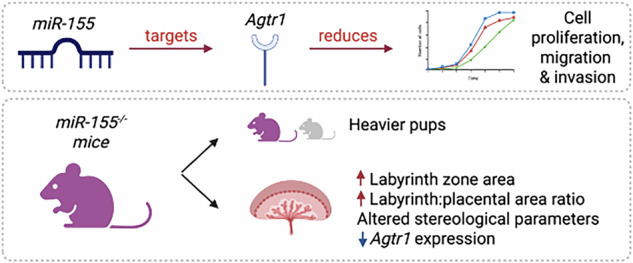

## Introduction

Placentation is a finely tuned process, relying on complex integrated genetic control. Post-transcriptional regulation by microRNAs (miRNAs) plays a role in this process [1]. miRNAs are short, non-coding RNA fragments that largely act by repressing mRNA translation. One miRNA that has been proposed to regulate placental development is *miR-155* [2–7]. *miR-155* is increased in placentae from pregnancies complicated by preeclampsia [8–10] and in vitro has been shown to inhibit trophoblast proliferation, migration, and invasion [6, 8]. Furthermore, pregnant mice with placental-specific *miR-155* overexpression had significantly reduced fetal and placental weights, with decreased trophoblast invasion and insufficient remodeling of uterine spiral arteries [10]. Together, these studies provide strong evidence that *miR-155* is a key regulator of placental development.

miRNAs can target multiple mRNA sequences, and *miR-155* has been demonstrated to target several factors that contribute to placental development, including Cysteine-rich angiogenic inducer 61 (*CYR61*) [11], cyclin D1 (*CCND1*) [12], Forkhead box O-3 (*FOXO3*) [8], and Mothers against decapentaplegic homolog 2 (*SMAD2*) [6]. Outside of pregnancy, *miR-155* has been shown to reduce the expression of the angiotensin II type 1 receptor gene (*AGTR1*) [13, 14], a known driver of placental development, and polymorphisms in the 3’-UTR of the *AGTR1* mRNA are known to inhibit *miR-155* binding [15]. Importantly, when human umbilical vein endothelial cells (HUVECs) isolated from healthy and preeclamptic pregnant women were transfected with a mature *miR-155* plasmid, luciferase assays confirmed the direct effect of *miR-155* on *AGTR1* as a target gene [16]. However, their interactions in the placenta and in regulating placental development are yet to be investigated.

The AT_1_R is the receptor for angiotensin (Ang) II and, therefore, a key component of the reninangiotensin system (RAS). Although the circulating RAS is well known for its roles in cardiovascular control and salt and water homeostasis, tissue RASs also exist, including a placental RAS that plays an important role in placental development [17]. In tissues, Ang II acts via the AT_1_R to stimulate proliferation, migration [18], and angiogenesis [18, 19]. In humans, the expression of a number of the genes and proteins of the placental RAS, including the AT_1_R, are highest in early gestation [17], supporting its role in early placental development. The importance of adequate AT_1_R expression in placental development was established by Walther et al., where a null mutation in the gene encoding AT_1_R was associated with much smaller and more poorly vascularised placentae, which adversely impacted fetal development [20]. Therefore, the potential effect of *miR-155* on *AGTR1* mRNA expression and how this affects placentation requires investigation.

In this study, we utilized mice with a null mutation in *miR-155* to examine the role of *miR-155* in placental development by studying placental morphology and stereology and fetal development. The effect of genetic ablation of *miR-155* on placental AT_1_R expression was also investigated as a key pathway in placental development. Given the importance of the placental renin-angiotensin system in controlling trophoblast proliferation [21, 22], we subsequently investigated the direct effects of *miR-155* on trophoblast proliferation, migration and invasion, in addition to AT_1_R expression using HTR-8/SVneo cells.

## Results

### Placental and fetal weights from *miR-155*^+/+^ (control) and *miR-155*^-/-^ mice

**Fig. 1:**
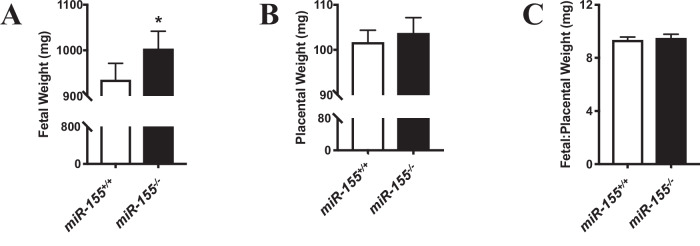
Fetal and placental weights from miR-155^-/-^ and miR-155^+/+^ mice at pc day 18.5. **A** Fetal and (**B**) placental weights, and **C** fetal to placental weight ratios in *miR-155*^-/-^ and *miR-155*^+/+^ (control) groups. Data are represented as mean ± SEM. n_*miR-155*(+/+)_ = 18 litters with 4–12 fetuses/placentae each to a total of 96. n_*miR-155*(-/-)_ = 18 litters with 1–5 fetuses/placentae each to a total of 86. *indicates a significant difference from controls (*p* < 0.05) as analyzed using a linear mixed model with random intercept accounting for maternal familial correlation.

On pc day 18.5, pups from *miR-155*^-/-^ dams had significantly greater fetal weights than pups from control dams (*p* = 0.003; Fig. 1A), but placental weights (Fig. 1B) and fetal to placental weight ratios (Fig. 1C) were not significantly different.

Numbers of embryos, viable embryos and resorptions were also assessed, however no significant differences were determined (Supplementary Fig. 2).

### Placental morphology in control and *miR-155*^-/-^ mice

**Fig. 2:**
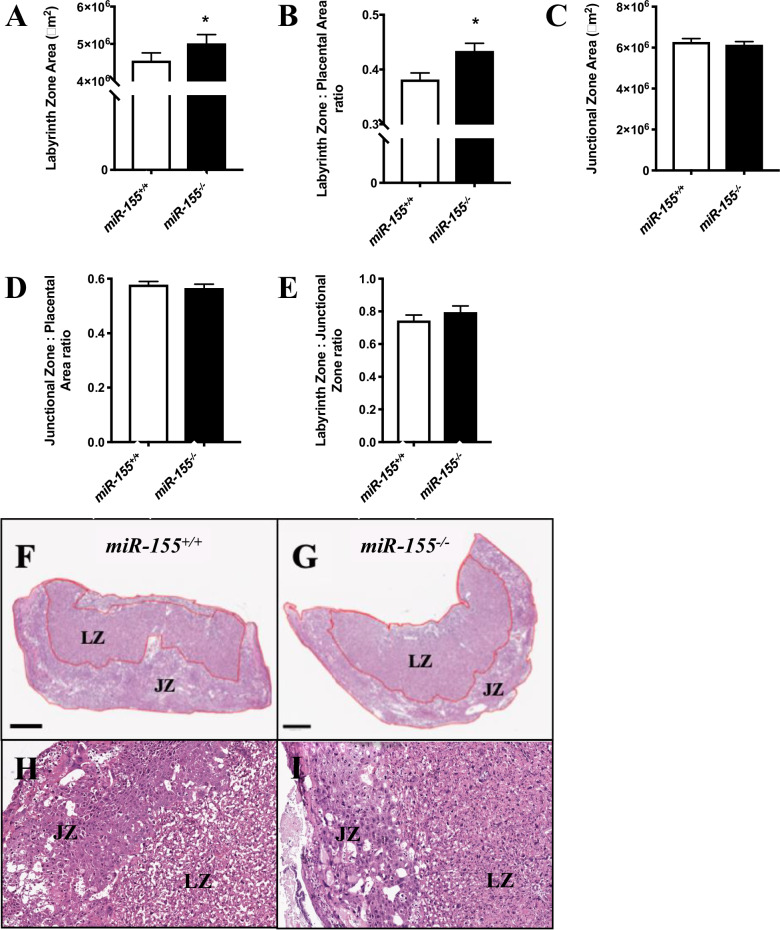
Morphological analyses of placentae from miR-155^-/-^ and miR-155^+/+^ mice at pc day 18.5. **A** Labyrinth zone area, **B** labyrinth zone to placental area ratio, **C** junctional zone area, **D** junctional zone to placental area ratio and **E** labyrinth zone to junctional zone area in placentae *miR-155*^-/-^ and control mice. **F** Labyrinth zone (LZ) compared with junctional zone (JZ) from the placenta of a control dam and **G** from the placenta of a *miR-155*^-/-^ dam. **H** Magnified (40X objective) images depicting trophoblast cell morphology and spiral artery remodeling in the placenta of a control dam and **I** from the placenta of a *miR-155*^*-/-*^ dam. Data are represented as mean ± SEM. n_*miR-155*(+/+)_ = 18 litters with 4–12 placentae each to a total of 96 (all placentae). n_*miR-155*(-/-)_ = 18 litters with 1–5 placentae each to a total of 86. * indicates a statistically significant difference from controls (*p* < 0.05).

Placentae from *miR-155*^-/-^ mice had a significantly larger labyrinth zone (*p* = 0.02; Fig. 2A) and labyrinth zone to placental area ratios (*p* = 0.04; Fig. 2B) compared with placentae from control mice. However, the area of the junctional zone (Fig. 2C), junctional zone to placental area ratio (Fig. 2D) and labyrinth zone to junctional zone ratio (Fig. 2E) were not different between control and *miR-155*^*-/-*^ mice. This is shown in representative images of placentae from control (Fig. 2F) and *miR-155*^-/-^ (Fig. 2G) mice.

### Stereological analysis of placental tissue

Placental morphology of *miR-155*^-/-^ and controls were evaluated using Merz grid stereological analysis (Table 1 and Supplementary Fig. 3). *miR-155*^-/-^ placentae displayed a significant increase in trophoblast volume density (*p* < 0.0001) and trophoblast weight (*p* < 0.0001) when compared with the control placentae. Trophoblast surface density (*p* = 0.0005) and total surface area of trophoblasts (*p* < 0.0001) were also significantly increased in *miR-155*^-/-^ placentae compared to controls. Interestingly, fetal capillary surface density was decreased in *miR-155*^-/-^ placentae (*p* = 0.0001) compared to controls, as was mean barrier thickness, a pseudo-measure of syncytiotrophoblast barrier thickness (*p* = 0.005).

**Table 1:** Stereological assessment of placental tissue.

		*miR-155*^+/+^	*miR-155*^-/-^
		*n* = *96*^*a*^	*n* = *86*^*b*^
Volume Density	TB	0.24 ± 0.05	**0.35** ± 0.03****
	FC	0.51 ± 0.06	0.50 ± 0.04
	MBS	0.25 ± 0.04	0.24 ± 0.02
Component Weight (g)	TB	0.02 ± 0.006	**0.03** ± 0.003****
	FC	0.05 ± 0.006	0.05 ± 0.004
	MBS	0.02 ± 0.004	0.02 ± 0.004
Surface Density (nm^2^/g)	TB	2202 ± 134.8	**2683** ± 71.44***
	FC	4303 ± 116.7	**3778** ± 53.19***
	MBS	1933 ± 40.1	1857 ± 60.83
Total Surface Area (μm^2^)	TB	1.08 ± 0.21	**1.48** ± 0.28****
	FC	2.15 ± 0.37	2.08 ± 0.31
	MBS	0.97 ± 0.18	1.02 ± 0.16
Mean Barrier Thickness			
(μm)		115.3 ± 21.56	**134.0** ± 17.94**

Data are presented as mean ± SEM. Data were analyzed by Two-way ANOVA: Šídák’s multiple comparisons test. *Denotes a significant difference compared with the control (*miR-155*^+/+^) group (**≤0.005, ***≤0.0005, ****≤0.0001). Values in bold are to highlight significant changes denoted by an asterisk (*).

*TB* trophoblasts, *FC* fetal capillaries, *MBS* maternal blood space.

^a^*n* = 18 litters with 4–12 fetuses and placentae each to a total of 96 (all placentae).

^b^*n* = 18 litters with 1–5 fetuses and placentae each to a total of 86.

### Effect of *miR-155* deficiency on uteroplacental vascular function

Given the alterations to placenta stereology, we next sought to determine the impact of *miR-155* deficiency on uterine and umbilical artery function in vivo. To achieve this, we undertook ultrasound biomicroscopy on pregnant *miR-155*^-/-^ and control mice on day 17.5 postcoitum. Analysis of uterine artery function showed that there were no differences in the resistance index or the pulsatility index between control and *miR-155*^-/-^ mice, both measures of uterine artery resistance to blood flow (Supplementary Fig. 4). Similarly, *miR-155* deficiency did not impact the umbilical artery resistance index and pulsatility index, however, there was a reduction in end diastolic velocity (*p* = 0.036) were observed in the umbilical arteries of *miR155*^-/-^ mice compared to those of control dams (Supplementary Fig. 5). Umbilical artery function was not correlated with fetal weight or fetal sex (data not shown).

### *miR-155* and AGTR1 mRNA, and AT1R protein expression, in control and *miR-155*^*-/-*^ mice

**Fig. 3:**
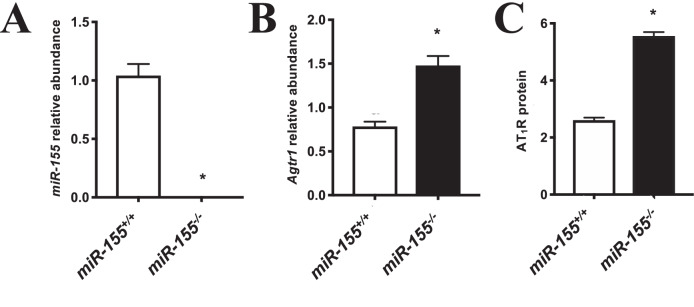
Expression of miR-155 and Agtr1 mRNA and AT_1_R protein in placentae from miR-155^-/-^ and control mice. **A** Expression of *miR-155*, **B** *Agtr1* mRNA and **C** AT_1_R protein levels in placentae from *miR*-*155*^-/-^ and control mice. Data are represented as mean ± SEM. *n* = 4–9 placentae/dam for ten dams total. * indicates a statistically significant difference to controls (*p* < 0.05).

The expression of miR-155 was absent in placentae from miR-155^-/-^ compared with control mice (*p* = 0.0003; Fig. 3A). *miR-155* targets several different mRNA transcripts that encode for proteins that are associated with placental development, including *Cyr61, Ccnd1, Foxo3*, and *Smad2*. Therefore, qPCR was used to assess the relative abundance of these mRNAs in placentae from *miR-155*^*-/-*^ compared with control mice, however no significant differences were observed between groups (data shown in Supplementary Fig. 6). Conversely, another known *miR-155* target, AT_1_R, was altered in *miR-155*^*-/-*^ mice placentae. Specifically, *Agtr1* mRNA and AT1R protein levels were significantly increased in placentae from *miR-155*^*-/*-^ compared with control mice (*p* = 0.01 and *p* = 0.004, respectively; Fig. 3B, C).

Representative full-length images of immunoblots are provided in Supplementary Fig. 7.

### Effect of *miR-155* on trophoblast proliferation in HTR-8/SVneo cells

**Fig. 4:**
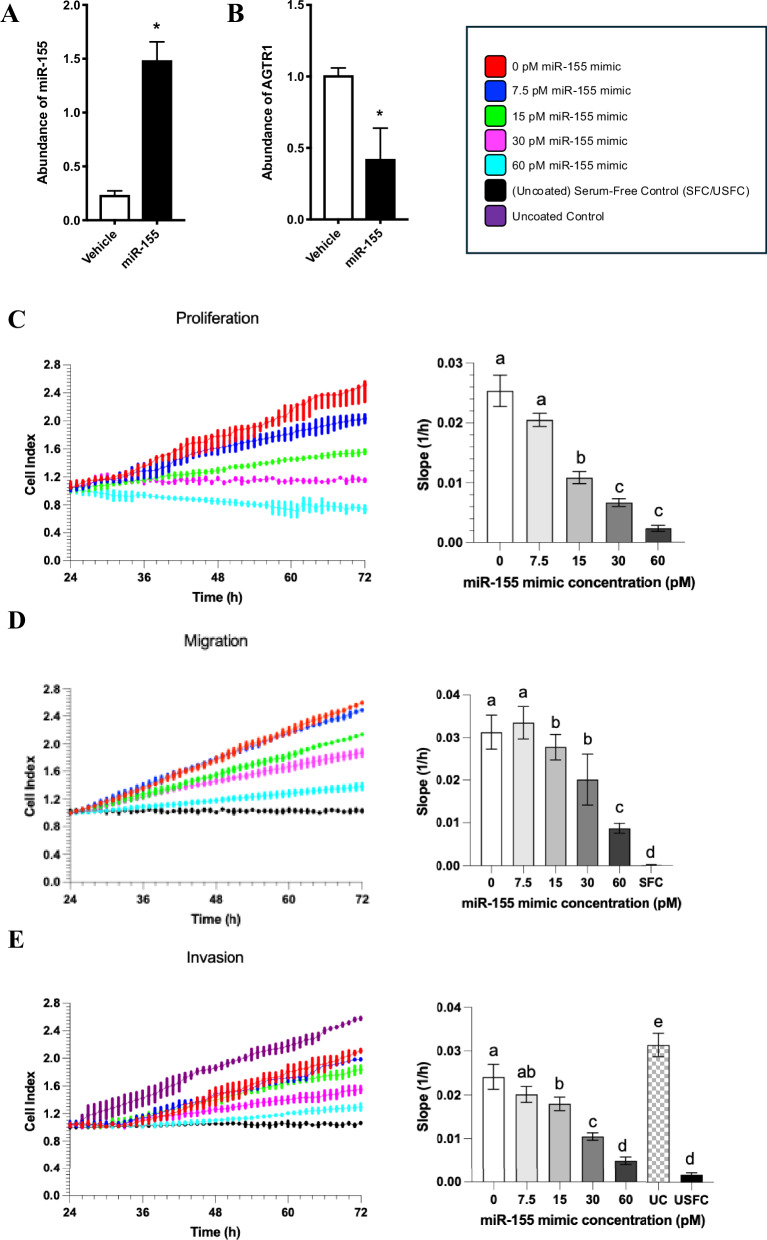
Abundance of miR-155 and AGTR1 mRNA, and the rate of cell proliferation in miR155 mimic-treated and vehicle-treated HTR-8/SVneo cells. **A** *miR-155* and **B** *AGTR1* mRNA expression in *miR-155* mimic-treated and vehicle-treated HTR-8/SVneo cells. **C** The rate of proliferation, **D** migration and **E** invasion of HTR-8/SVneo cells transfected with different doses of a *miR-155* mimic. The slopes of the lines indicate HTR8/SVneo cell proliferation, migration and invasion, respectively, over 72 h. Control (red), 7.5 pM mimic (blue), 15 pM mimic (green), 30 pM mimic (magenta), 60 pM mimic (cyan). Graphs **D**, **E** include an uncoated serum-free control (black); graph **E** includes an uncoated control (purple). Data are presented as mean ± SEM. *n* = 3 experiments, each in triplicate. * indicates a statistically significant difference to the vehicle control (*p* < 0.05). The same letter above the bars indicates that groups are not different from each other. A different letter above the bars indicates that groups are different (all *p* < 0.05).

In vitro experiments were employed to further investigate the effects of *miR-155* on trophoblast cell function. HTR-8/SVneo cells treated with a *miR-155* mimic had a significantly increased abundance of *miR-155* (*p* = 0.003; Fig. 4A) and significantly decreased abundance of *AGTR1* mRNA (*p* = 0.005; Fig. 4B) compared with the scrambled control. Treatment with 15, 30 and 60 pM of *miR-155* mimic significantly decreased trophoblast proliferation compared with the control (*p* = 0.029, *p* = 0.005, *p* < 0.001, respectively; Fig. 4C). Proliferation was also significantly decreased at 15, 30 and 60 pM compared with 7.5 pM (*p* = 0.035, *p* = 0.047, *p* < 0.001, respectively). The cell index trajectory displays the gradient (slope) as an indication of cell activity.

As described in Supplementary Fig. 1, no significant change in proliferation was detected between the negative control (0 pM scrambled mimic + Lipofectamine) and all concentrations of the scrambled control, confirming that our results are *miR-155* sequence-specific.

### Effect of *miR-155* on trophoblast migration in HTR-8/SVneo cells

Treatment with 15, 30 and 60 pM of *miR-155* mimic significantly decreased trophoblast migration compared with the control (*p* = 0.001, *p* < 0.001, *p* < 0.001 respectively; Fig. 4D). Migration was also significantly decreased at 15, 30, and 60 pM compared with 7.5 pM (*p* = 0.013, *p* = 0.002, *p* < 0.001, respectively). Migration was significantly decreased at 60 pM compared with 15 and 30 pM (all *p* < 0.001). Migration was significantly decreased in SFC compared with all treatment conditions, i.e., 0, 7.5, 15, 30, and 60 pM mimic (all *p* < 0.001).

### Effect of *miR-155* on trophoblast invasion in HTR-8/SVneo cells

Treatment with 15, 30, and 60 pM of *miR-155* mimic significantly decreased trophoblast invasion compared with the control (*p* = 0.048, *p* = 0.001, *p* < 0.001 respectively; Fig. 4E). Invasion was also significantly decreased at 30 and 60 pM compared with 7.5 pM (*p* = 0.011, *p* < 0.001, respectively). Invasion was significantly decreased at 30 pM compared with 15 pM (*p* = 0.038) and at 60 pM compared with 15 and 30 pM (all *p* < 0.001). Invasion was significantly decreased in USFC compared with 0, 7.5, 15, and 30 pM mimic and USFC (all *p* < 0.001) and was significantly increased in UC compared with all conditions, i.e., 0, 7.5, 15, 30, and 60 pM mimic (all *p* < 0.001).

## Discussion

We have shown that *miR-155* modulates placental cell function and development and that maternal and placental null mutation in *miR-155* disrupts placental morphology by altering labyrinth and junctional zone development. We propose that this occurs, at least in part, due to the inhibitory action of *miR-155* on *Agtr1* mRNA expression in turn causing reduced AGTR1 synthesis.

The impact of the *miR-155* null mutation was readily observed when placental morphology was analyzed. In *miR-155*^-/-^ placentae, there was a significant increase in the labyrinth zone area, as well as the labyrinth zone to total placenta area ratio. The labyrinth zone is closest to the fetus and is considered to be the functional zone of the placenta where nutrient, gas and waste exchange occurs [23]. This increase in labyrinth zone size suggests that there was increased trophoblast differentiation, resulting in increased substrate transfer capacity of the placenta and, in turn, an increased ability to accelerate growth and sustain a larger fetus [24]. Placentae from *miR-155*^-/-^ mice had higher trophoblast component weight in conjunction with increased trophoblast surface area when assessed by stereology, although placental weight did not significantly change between *miR-155*^-/-^ and control mice. Compared to placentae from control mice, placentae from *miR-155*^-/-^ mice also had higher trophoblast volume density and higher trophoblast surface density, indicating increased numbers of trophoblasts and that a greater proportion of the placenta area is covered by trophoblasts, respectively. Placentae from *miR-155*^-/-^ mice also had increased mean barrier thickness (an indication of syncytiotrophoblast barrier thickness) compared with controls. These results are in accordance with a previous study by Wang et al. [10], who showed that *miR-155* overexpressing placentae exhibited a smaller labyrinth layer, and a disorganized arrangement of trophoblast cells [10]. Wang et al. also demonstrated however that the *miR-155* overexpressing placentae displayed decreased placental vascular networks. This contrasts with the current study, where placentae from *miR-155*^-/-^ showed decreased fetal capillary surface density. Thus, further investigation into the role of *miR-155* in placental vascularization is warranted.

Indeed, while *miR-155* deficiency did not affect uterine artery function, it did have some impact on umbilical artery function. Umbilical artery Doppler velocimetry assesses resistance to fetal blood flow within the placenta. Various conditions can compromise the placental vasculature, leading to decreased, absent, or reversed end-diastolic velocity in the umbilical artery [54]. We observed that maternal *miR-155* deficiency was associated with decreased umbilical end-diastolic velocity compared to controls, however, since this change was not accompanied by fetal growth restriction or alterations in the resistance or pulsatility index, the clinical significance of this finding may be limited [53, 54].

Together, these changes in placental structure are consistent with our observation of larger fetuses in *miR-155*^-/-^ compared with controls, as well as literature indicating a positive correlation between placental volume and fetal growth [25]. Indeed, at the same gestational age, *miR-155*^-/-^ mice did have larger fetuses than controls, without any concomitant alteration to other fetal parameters. Given that we established the inhibitory action of *miR-155* on AT_1_R expression, these findings correlate with the findings of Walther et al., where placental AT_1_R knockout resulted in smaller placentae and fetuses [20]. Our results are further corroborated by the findings of Wang et al., who observed that placenta specific *miR-155* overexpression in pregnant dams produced smaller and significantly fewer pups [10].

While our data point to an association between *miR-155* regulation of AT_1_R expression in influencing placental development, it is important to acknowledge that the effects of *miR-155* deficiency on placental development may also be exacerbated by altered maternal immune parameters. We have previously shown profound changes to the maternal immune environment in pregnant *miR-155* deficient female mice [26]. Maternal *miR-155* was shown to be required for expansion of regulatory T cells to mediate robust pregnancy tolerance and support fetal development in mice [26]. Additionally, Treg cells contribute to the control of maternal vascular function during pregnancy [55, 56] further highlighting the potential link between *miR-155* deficiency, immune cells and placental function. However, *miR-155* driven immune alterations likely play a less essential role in the current study as pregnancies were generated by syngeneic matings, which are less dependent on maternal immune tolerance than the allogeneic mating model used in our previous work [26]. This may explain why in the current study, *miR-155* deficiency leads to an increase in fetal weight, despite the fact that compromised Treg cell number and function in pregnancy is associated with pregnancy complications such as fetal growth restriction and preeclampsia [57, 58, 59].

Adequate trophoblast proliferation is essential for placental growth. We showed that treatment with a *miR-155* mimic decreased HTR-8/SVneo cell proliferation in a dose-dependent manner, consistent with previous studies reporting that *miR-155* decreases cell proliferation [5, 27]. Recent studies have confirmed that transfection of HTR-8/SVneo cells with a *miR-155* mimic also results in reduced trophoblast migration and invasion [6]; likewise, inhibition of *miR-155* expression in HTR-8/SVneo and JEG3 cell lines resulted in increased proliferation, migration and invasion [28]. This is coherent with our findings that *miR-155* deficient placentae are more efficient through larger labyrinth zone area and increased trophoblast component weight and volume density, although we did not detect a significant difference in placental weight between the two groups. Furthermore, we demonstrated that *miR-155* mimic treatment significantly reduced the migratory and invasive capacity of HTR-8/SVneo cells in a dose-dependent manner. As these processes are critical for extravillous trophoblast differentiation and successful uterine implantation [29], our findings suggest that *miR-155* may impair placental development by disrupting these key pathways.

The actions of *miR-155* are complex, as it is known to regulate many targets in addition to the gene encoding AT_1_R. Within the placenta, studies have associated *miR-155* expression with downregulation of cysteine-rich protein 61 (CYR61), which is an important angiogenic regulating factor during pregnancy [11]. Importantly, CYR61 is essential for placental vascular integrity [30] and induces expression of vascular endothelial growth factor (VEGF) [31] and is shown to be decreased in preeclampsia [32]. Indeed, in a rat model of preeclampsia, *miR-155* was increased, and VEGF decreased compared with controls [5]. *miR-155* has also been implicated in the suppression of the PTEN 30-untranslated region, leading to reduced AP-1/NF-kB pathway activity [7]. *miR-155* downregulates cyclin D1 [12], a critical regulator of cell cycle progression and transcriptional co-regulator, along with *Smad2* [6], a signal transducer that interacts with the TGF-β receptors, and FOXO3 [8], which is suspected to function as a trigger for cellular apoptosis. Despite these reported interactions between *miR-155* and targets that are likely involved in placental development and function, we found no evidence that the mRNA expression of these targets was altered in the placentae of *miR-155*^-/-^ mice (Supplementary Fig. 6). We acknowledge that *miR-155* may regulate not only these targets, but also other targets, post-transcriptionally, and that the fetal and placental phenotypes observed are likely the result of combined effects on multiple pathways. However, the substantial upregulation of AT_1_R expression in *miR-155*^-/-^ placentae supports our hypothesis that *miR-155* is at least partly acting through the AT_1_R to induce its effects.

Notably, *miR-155* directly interacts with the 3’-UTR of *AGTR1* mRNA [33], and the interaction between *miR-155* and *AGTR1* has been established in patient-derived HUVECs from preeclamptic and healthy pregnancies [16]. Furthermore, the silent polymorphism +1166A/C in the 3’-UTR of *AGTR1* has been associated with hypertension and cardiovascular complications, with expression of the mutant allele resulting in decreased *miR-155* expression, likely due to difficulty binding to the mRNA. AT_1_R protein expression was significantly increased and positively correlated with systolic and diastolic blood pressure in pregnancy [34]. The current study provides robust evidence that *miR-155* negatively regulates placental development, and proposes a novel mechanism involving its interaction with AT_1_R. Specifically, we observed ~2-fold increased *Agtr1* mRNA expression and almost 3-fold increased AT_1_R protein in *miR-155*^-/-^ placentae, in the absence of changes at the mRNA level for other targets or *miR-155*, indicating an association between *miR-155* and AT_1_R expression as well as placental growth. Further evidence for the *miR-155*/AT_1_R axis exerting control over placental cell growth was seen using treatment with a *miR-155* mimic, which reduced *AGTR1* mRNA expression by approximately half, also had a dose-dependent negative effect on HTR-8/SVneo cell proliferation.

It is worthy to note that in rodents AT1R exists as two isoforms, *Agtr1a* and *Agtr1b*, which share a high degree of sequence similarity. Although *Agtr1a* is the predominant isoform expressed in the placenta [35], the primers used in our qPCR assay do not distinguish between the two isoforms. Therefore, the expression data presented here likely reflect combined levels of *Agtr1a* and *Agtr1b*. Future studies could employ isoform-specific approaches to further delineate the contribution of each receptor subtype to placental development. Furthermore, luciferase reporter assays or mutation of the *miR-155* seed sequence binding site within *Agtr1a* and *Agtr1b* mRNA isoforms would provide definitive mechanistic confirmation, instead of the associations reported here which indicate that *miR-155* may directly and/or indirectly regulate AGTR1 expression and contribute to placental development and pathology. However, these experiments are beyond the scope of the current study.

This study confirms the importance of adequate Ang II/AT_1_R signaling in placentation and reinforces the findings of Delforce et al., showing that specific inhibition of the AT_1_R by losartan inhibits expression of the angiogenic/proliferative trophoblast phenotype [36]. This phenotype is highly correlated with the expression of pro-angiogenic factors and cell viability [36]. The binding of Ang II to the AT_1_R has also been implicated in cytotrophoblast secretion of many hormones essential for healthy pregnancy, including placental lactogen, human chorionic gonadotropin and pregnancy-specific glycoprotein [37], as well as estradiol [38]. As mentioned previously, AT_1_R knockout in the placenta severely impedes placental growth and vasculogenesis [20]. Even intrauterine growth restriction is associated with reduced placental *AGTR1* mRNA and AT_1_R protein [39], which is consistent with our results as *miR-155*^-/-^ placentae expressed significantly higher levels of AT_1_R mRNA and protein, and produced larger fetuses.

Clinically, our findings have potential translational relevance, as *miR-155* dysregulation has been associated with several human pregnancy complications. Significantly lower levels of *miR-155* are recorded in first-trimester peripheral blood from women who later experienced recurrent pregnancy loss, suggesting that early *miR-155* dysregulation may compromise pregnancy maintenance [40]. In contrast, elevated placental *miR-155* expression has been observed in patients with preeclampsia, where it was shown to directly repress *AGTR1* expression [10]. Other studies have similarly reported upregulation of *miR-155* in preeclamptic placentae ([4, 28, 41–43]), and *miR-155* has also been implicated in the pathogenesis of intrauterine growth restriction through its impact on trophoblast proliferation and invasion [6] and as a circulating biomarker of small for gestational age pregnancies [44]. These findings support a model in which precise regulation of *miR-155* is essential for normal placental development and function, and implicate *miR-155* as a potential biomarker or therapeutic target in pregnancy disorders. Indeed, Wang et al. [10] showed that antagomir-155 could eliminate the preeclampsia-like manifestations in pregnant mice in which *miR-155* is overexpressed in the placenta [10]. Notably, this study was intended as mechanistic proof-of-concept, and while *miR-155* may represent a useful biomarker, its direct therapeutic targeting would require caution due to the risks of disrupting renin–angiotensin signaling in pregnancy [45].

Indeed, the differential expression of *miR-155* in maternal blood from women experiencing pregnancy complications raises the possibility that it may function as a cell-free or exosomal signal during pregnancy. Indeed, several studies have proposed *miR-155* as a potential noninvasive biomarker for various pregnancy complications ([2, 4, 10]). While our study focused on placental expression, future investigations could explore whether *miR-155* is released into the maternal circulation in response to placental stress or dysfunction, and whether it contributes to intercellular communication between the placenta and maternal tissues. This highlights the broader physiological relevance of *miR-155* beyond its local effects within the placenta.

Overall, we have shown that culturing trophoblast cells with a *miR-155* mimic decreased both *AGTR1* mRNA and AT_1_R protein and impaired trophoblast proliferation, migration and invasion. This was supported by our finding that placentae from *miR-155*^-/-^ dams had increased levels of *Agtr1* mRNA and AT_1_R protein accompanied by increased fetal weights and an increase in trophoblasts and labyrinth zone area. The study therefore provides compelling evidence that *miR-155* impacts placental morphology and functional capacity, resulting in altered fetal growth. We conclude that the effects of *miR-155* on placental development and fetal growth are at least partly due to its regulation of AT_1_R expression.

## Methods

### Animal work

*Bic*/*miR-155* (*miR-155*^-/-^) mice on a C57Bl/6 background [26] generated by Prof Klaus Rajewsky (CBR Institute for Biomedical Research, Harvard Medical School) [46] were purchased from The Jackson Laboratory (Bar Harbor, ME, USA, Stock No: 007745) and bred in-house at the University of Adelaide. C57Bl/6J (B6; *miR-155*^+/+^) wildtype control female mice were purchased from Animal Resource Centre, Perth. All mice were co-housed in specific pathogen-free conditions at the University of Adelaide Medical School Animal House on a 12-h light-dark cycle and were administered food and water *ad libitum*.

Experimental females were 8–12 weeks, and males were 10 weeks to 12 months in age.

For mating experiments, *miR-155*^*+/+*^ and *miR-155*^*-/-*^ females were mated with the same genotype males, and 1–2 adult female mice were caged with one male for mating. Mice were checked for a vaginal plug daily (checked between 8 am and 10 am), and females on the day of plug detection were designated post coitum (pc) day 0.5. Mated females were housed in groups of 1–4 females per cage. No randomization or blinding was used.

The number of mice per experiment is listed in the corresponding Figure legend.

### Ultrasound

On pc day 18.5, both uterine and umbilical artery function were assessed using an MX550D transducer probe on a Vevo 3100 ultrasound biomicroscope (VisualSonics®, ON, Canada), as described previously [55]. Briefly, mice were anaesthetised with isoflurane (5% induction, 1.5% maintenance, in medical air) and Doppler waveforms were acquired from uterine arteries near the lateral inferior margin of the utero-cervical junction and from the umbilical arteries of 3–4 fetuses per dam. During the ultrasound, the location of each fetus was marked on the skin of the abdomen of the anesthetised dam using a permanent marker. Immediately following the ultrasound, at postmortem, the maternal abdominal wall was carefully opened without disturbing the uterus, and corresponding marks were made on the uterine surface to identify the studied fetuses. These fetuses were dissected and weighed first to ensure accurate identification before processing the remaining fetuses. Each waveform was analyzed in triplicate, following previously established methods. Peak systolic velocity (PSV), end-diastolic velocity (EDV), time-averaged velocity (TAV) and heart rate averages were obtained from a minimum of three consecutive cardiac cycles. Resistance index (RI = (PSV − EDV)/PSV) and pulsatility index (PI = (PSV − EDV)/TAV) were calculated. Following Doppler imaging, dams were humanely killed by cervical dislocation. Each viable fetus was dissected from the amniotic sac and umbilical cord, and the fetuses and placentae were weighed. Placental tissues were either cryo-frozen or fixed in 10% formalin and washed in phosphate-buffered saline before embedding in paraffin.

### RNA extraction and DNase treatment

Total RNA was extracted from crushed cryo-frozen placental tissue using TRIzol Reagent

(Thermo Fisher Scientific, Waltham, MA, USA), according to the manufacturer’s instructions.

Tissues (~0.15 g) were homogenized with 1.5 mL of TRIzol in a Precelleys24 homogenizer (5000 RPM, 2 × 30 s, then 1 × 20 s, Thermo Fisher Scientific). DNase I treatment (Qiagen GMBH, Hilden, Germany) was performed on all samples. The integrity of the total RNA and miRNAs were examined by gel electrophoresis and quantified using the Nanodrop 2000 (Thermo Fisher Scientific, data not shown [47]). Samples were used for further analysis if the 260:280 and 260:230 nm ratios were 1.8–2.1 and 2.0–2.2, respectively.

### miRNA analysis

Expression of *miR-155* was measured by qPCR [21]. Total RNA (5 ng) samples underwent reverse transcription to cDNA (TaqMan miRNA Reverse Transcription Kit and probes, Assay ID #002571 for human *miR-155*, #002571 for mouse *miR-155*, #001094 for *RNU44* and # 002491 for *miR-20a*; Thermo Fisher Scientific) according to the manufacturer’s instructions. Samples then underwent quantitative PCR (qPCR) using TaqMan Universal PCR master mix, according to the manufacturer’s instructions (Thermo Fisher Scientific). Data was generated using a 7500 Real-Time PCR System (Thermo Fisher Scientific). The expression of *miR-155* was determined by calculating 2^−ΔΔCT^ using *RNU44* (a highly conserved small nucleolar RNA in the growth arrest-specific 5 transcript) as the human reference gene and *miR-20a* as the mouse reference gene.

### Quantitative reverse transcriptase polymerase chain reaction (qPCR)

All RNA samples underwent reverse transcription to generate cDNA according to the manufacturer’s instructions (Superscript III First-Strand Synthesis for RT, Thermo Fisher Scientific). Total RNA was spiked with a known amount of Alien RNA (Stratagene; 10^7^ copies per µg of total RNA). qPCR was performed in an Applied Biosystems 7500 Real-Time PCR System using SYBR Green for detection. Each reaction mixture contained 5 μL of SYBR Green PCR master mix (Applied Biosystems), primers, 10 ng cDNA, and water to 10 μL. Messenger RNA abundance was calculated as described previously, using the 2^–ΔΔCT^ method and expressed relative to Alien mRNA and a calibrator cDNA (prepared from pooled mouse kidney tissue) [17]. Primer sequences for *Agtr1* (primers capture expression of both *Agtr1a* and *Agtr1b* isoforms) [48]; *Foxo3* F: CTCTCAGGCTCCTCACTGTA, R: ATGAGTTCACTACGGATGAT; *Ccnd1* F: GAACAGACAAGCACATTAATAGA, R: GCTTCAGTTCATGAGTCTTATTCC; *Cyr61* F: CGAGTTACCAATGACAACCCAG, R: TGCAGCACCGGCCATCTA, *Smad2* F: GAGTGTGGATTGTTACCTTTG, R: CTCAACTCTCTGGTAGTGGTA.

### Protein extraction

Protein was isolated from tissues using a radioimmunoprecipitation assay buffer (RIPA, 50 mM Tris-HCl, 150 mM NaCl, 1 mM EDTA, 1% Triton X–100, 1% sodium dodecyl sulfate, SDS) supplemented with a Pierce Halt™ complete protease inhibitor cocktail tablet (Thermo Fisher Scientific), in a ratio of 250 μL buffer to 200 mg of tissue. Samples were then homogenized using the Precelleys24 homogenizer (5000 RPM, 2 × 30 s, then 1 × 20 s, Thermo Fisher Scientific, Waltham, MA, USA) every 10 min across a 30 min period and cooled on ice in between. Protein levels were measured using the Pierce BCA Protein assay kit (Thermo Fisher Scientific) according to the manufacturer’s instructions.

### Immunoblotting

Immunoblotting was performed as described previously [48, 49]. Briefly, samples were loaded into Bis-Tris methane 4–12% gels in duplicate before electrophoresis (Thermo Fisher Scientific). Proteins were then transferred to a polyvinylidene difluoride (PVDF) membrane (Thermo Fisher Scientific) using the wet sandwich method immersed in Tris Buffered Saline + Tween20 (TBST) transfer buffer. The PVDF membrane was then completely dried and re-activated before immunodetection. The membrane was rocked in a blocking solution (5% BSA, 5% skim milk in Tris buffered saline (TBS)) for 2 h on a rocker at 22 °C. The primary antibody solution was then added (1:1000 dilution, Abcam, Cambridge, United Kingdom, #ab18801), and samples were incubated at 4 °C overnight. The secondary anti-rabbit antibody solution was added (1:5000 dilution, Millipore, Burlington, MA, USA; #12-348) and incubated at 22 °C on a rocker for 1 h. Membranes were rinsed before signal detection using an Amersham ECL detection kit and Amersham Imager 600 (both GE Healthcare, Chicago, IL, USA). Membranes were then dried and stripped using 0.2 M NaOH and, using a rabbit polyclonal anti-β-actin antibody (1:5000 dilution, Abcam; ab8227), were probed to detect β-actin for normalization. The ratio of the protein of interest to β-actin was averaged for duplicate lanes, and differences between blots were corrected using an internal control (a pooled mouse kidney sample). ImageJ software was used for assessment.

### Morphometric analysis

The mid-sagittal placental labyrinth and junctional zones were morphometrically analyzed, as previously described [50]. Briefly, tissue sections were stained with haemotoxylin and eosin and mounted before imaging. QuPath software [51] was used to measure the cross-sectional areas of the labyrinth, junctional zone and total placenta. From these measurements, ratios of labyrinth to junctional zone, labyrinth to total placenta and junctional zone to total placenta were calculated.

### Placental stereological analysis

Immunofluorescent dual labeling was performed as previously described [50]. Briefly, placental tissues were incubated with both mouse anti-cytokeratin (1:50 dilution, Agilent Technologies, Glostrup, Denmark; #M7018) and rabbit anti-vimentin (1:250 dilution, Abcam; #ab92547) primary antibodies overnight at 4 °C in 1% BSA-PBS. Following PBS-T washes, sides were incubated with both goat anti-mouse IgG H&L Alexa Fluor® 594 (1:500 dilution, Abcam; #ab150116) and goat anti-rabbit IgG H&L Alexa Fluor® 488 (1:500 dilution, Abcam; #ab150077) secondary antibodies, for 1 h at RT in 1% BSA-PBS. DAPIProLong Gold Antifade solution (Thermo Fisher Scientific) was applied before imaging at 20× magnification using an Axio Imager M2 microscope (Carl Zeiss AG, Jena, Germany) and analyzed using Zen imaging (Carl Zeiss AG) and ImageJ software (National Institutes of Health).

Immunofluorescent dual labeling was performed following established and widely accepted methodologies for quantifying placental architecture and blood space composition, as described in multiple prior studies ([23, 24, 50, 52]). To determine the proportion of placental trophoblasts, fetal capillaries, and maternal blood space, placental tissue dual labeled for cytokeratin and vimentin (as described above) underwent stereological analysis. The proportion of each component was quantified using point counting on an isotropic L-36 Merz grid using ImageJ imaging software. To ensure objectivity, uniform random sampling was employed to collect eight fields of view at 20x magnification per placental tissue.

The volume densities (V_d_) of placental trophoblasts, fetal capillaries, and maternal blood space, defined as the proportion of the reference volume (i.e., placental tissue) occupied by each component, were calculated using the following formula:

$$\mathrm{Volume}\,\mathrm{Density;}\,{{\rm{V}}}_{{\rm{d}}}\,=\,{{\rm{P}}}_{{\rm{a}}}/{{\rm{P}}}_{{\rm{T}}}$$

Where P_a_ is the total number of points that have fallen on the component of interest, and P_T_ is the total number of points applied to the image [24, 52].

The component weights (C_W_), representing the total mass of each placental component, the following formula was used:

$$\mathrm{Component}\,\mathrm{Weight;}\,{{\rm{C}}}_{{\rm{W}}}\,=\,{{\rm{V}}}_{{\rm{d}}}\,{\rm{x}}\,\mathrm{total}\,\mathrm{placental}\,\mathrm{weight}$$

The surface density (S_v_) of placental trophoblasts, fetal capillaries, and maternal blood space, quantifying the amount of surface area per unit of reference volume, were calculated using the following formula:

$$\mathrm{Surface}\,\mathrm{Density;}\,{{\rm{S}}}_{{\rm{v}}}\,=\,2\,{\rm{x}}\,{{\rm{L}}}_{{\rm{a}}}/{{\rm{L}}}_{{\rm{T}}}$$

Where L_a_ is the number of intercepts of the applied lines with the components of interest, and

L_T_ is the total length of the lines applied to the section [24, 52].

The total surface area (S_T_), which represents the full surface area for each component within the placental labyrinth zone, was determined by the equation:

Total Surface Area; S_T_ = S_v_ × placental weight × labyrinth proportion

The mean barrier thickness (B_T_), reflecting the average thickness of the syncytiotrophoblasts layer between the maternal and fetal circulations, was calculated using the following equation:

$$\mathrm{Barrier}\,\mathrm{Thickness;}\,\mathrm{BT}\,=\,{{\rm{V}}}_{{\rm{d}}}/{{\rm{S}}}_{{\rm{v}}}$$

Where V_d_ and S_v_ are the trophoblast volume density and trophoblast surface density, respectively [24, 52].

To assess reproducibility, observations were repeated on sections randomly throughout the analysis. The variation observed was less than 5%.

### Cell culture

HTR-8/SVneo cells (an immortalized first-trimester trophoblast cell line provided by Professor Charles Graham, Queens University, Ontario, free of mycoplasma) were cultured at 37 °C with 5% CO_2_ in RPMI1640 (Danaher Corporation, Washington, D.C., USA) supplemented with 10% heat-inactivated FCS (ISAFC Biosciences) and 1% L-glutamine (Thermo Fisher Scientific). Three separate cultures of HTR-8/SVneo cells were made, and from each of these three cultures, three sets of 2 × 105 cells from passages 10–20 were plated (*n* = 9). After 48 h, 30pM of the miRVana *miR-155* mimic (Thermo Fisher Scientific) was transfected into each well, using Lipofectamine (Thermo Fisher Scientific) as the transfection vector. To confirm a sequence-specific response, other wells were treated with Lipofectamine alone (negative control) or with the addition of a scrambled control (where nucleotides are scrambled in a random order to confirm sequence order is specific) at concentrations of 7.5, 15, 30 and 60 pM; data supplied in Supplementary Fig. 1. Cells and culture media were individually collected and snap frozen in liquid nitrogen, then stored at −80 °C before RNA analysis.

### Proliferation analysis

Fifty μL of cell culture medium was added to each well of an xCELLigence E-plate 16 (ACEA Biosciences Inc., San Diego, CA) and allowed to equilibrate at room temperature for 30 min. A background reading in the xCELLigence Real-Time Cell Analysis Multi Plate (RCTA MP) system was then carried out. 1 × 104 HTR-8/SVneo cells from passages 10–20 (cultured as above) were plated in each well with an additional 100 μL of incubation medium and again allowed to equilibrate for 30 min at room temperature. 24 h after cell plating, *miRNA-155* miRVana mimic, scrambled mimics or vehicle (Lipofectamine) were added to each well at 0, 7.5, 15, 30 or 60 pM. As the proliferation plates have gold microelectrodes on the bottom of each well, the proliferation of cells impedes electrical conductance. Cell index was generated as a measure of the electrical resistance as cells proliferated. The cell index was measured every 30 min for 48 h in the xCELLigence RCTA MP system. After 48 h of incubation with treatment, data were collected and analyzed. The rate of proliferation was determined by measuring the slope of the line (cell index) over time.

### Migration analysis

For cell migration analysis, an xCELLigence CIM-Plate 16 (ACEA Biosciences Inc., San Diego, CA) was used containing two chambers. 160 μL of incubation medium, with 10% FBS, was added to each well of the lower chamber, excepting a serum-free control (SFC). 50 μL of serum-free media was added to each well of the upper chamber, and the CIM-plate was equilibrated for 1 h at 37 °C, before a background measurement was taken using the RTCA software (Agilent, Santa Clara, CA, USA). A total of 3 × 10^4^ HTR-8/SVneo cells were plated in each well of the upper chamber before equilibrating for 30 min at room temperature. CIM plates were placed in the RTCA DP cradle and cell index readings were taken every 30 min for 72 h using the RTCA software (Agilent, Santa Clara, CA, USA). 24 h after cell plating, *miRNA-155* miRVana mimic, scrambled mimics or vehicle (Lipofectamine) were added to each well at 0, 7.5, 15, 30 or 60 pM.

### Invasion analysis

For cell invasion analysis, an xCELLigence CIM-Plate 16 (ACEA Biosciences Inc., San Diego, CA) was used containing two chambers. 160 μL of incubation medium, with 10% FBS, was added to each well of the lower chamber. 20 μL of Matrigel (1:20 ratio with serum-free incubation medium) pre-coated each well of the upper chamber prior to addition of 50 μL of serum-free incubation medium. Controls included 2 wells without Matrigel coating (“uncoated controls”; UC) and 2 wells without Matrigel coating or serum in the lower chamber (“uncoated serum-free control”; USFC). The CIM-plate was then equilibrated for 1 h at 37 °C, before a background measurement was taken using the RTCA software (Agilent, Santa Clara, CA, USA). A total of 3 × 10^4^ HTR-8/SVneo cells were plated in each well of the upper chamber before equilibrating for 30 min at room temperature. CIM plates were placed in the RTCA DP cradle and cell index readings were taken every 30 min for 72 h using the RTCA software (Agilent, Santa Clara, CA, USA). 24 h after cell plating, miRNA-155 miRVana mimic, scrambled mimics or vehicle (Lipofectamine) were added to each well at 0, 7.5, 15, 30, or 60 pM.

### Statistical analyses

Statistical analysis was undertaken using SPSS Statistics. Where multiple placentae or pups per mother were assessed, a linear mixed model with random intercept accounting for maternal familial correlation was used. Where a single placenta from each mother was assessed, a MannWhitney test was used. Stereology was analyzed by GraphPad Prism 8. Stereological analysis was statistically validated using Two-way ANOVA and Šídák’s multiple comparisons test, with variation between measurements assessed via Kruskal–Wallis: Dunn’s multiple comparisons test. All graphs were generated using GraphPad Prism 8. Differences between groups were considered significant for *p* < 0.05.

### Ethics

All experimental protocols were approved by the University of Adelaide Animal Ethics Committee (approval number: M-2014-023, M-2016-009), using methods in accordance with the Australian code for the care and use of animals for scientific purposes.

## Supplementary information

Supplementary Figure 1

Supplementary Figure 2

Supplementary Figure 3

Supplementary Figure 4

Supplementary Figure 5

Supplementary Figure 6

Supplementary Figure 7

## Data Availability

The authors declare that the data supporting the findings of this study are available within the paper and its Supplementary Information files. Should any raw data files be needed in another format they are available from the corresponding author upon reasonable request.
