# Supplementary material for: miR-155 suppresses angiotensin II type 1 receptor synthesis during placental morphogenesis
Source: Cell Death Discov. 2025 Dec 24;12:49. doi: 10.1038/s41420-025-02892-0 (PMC12847812; doi:10.1038/s41420-025-02892-0)
Supplement: Supplementary file 1 — Supplementary Figure 1 [file 41420_2025_2892_MOESM1_ESM.docx]

**
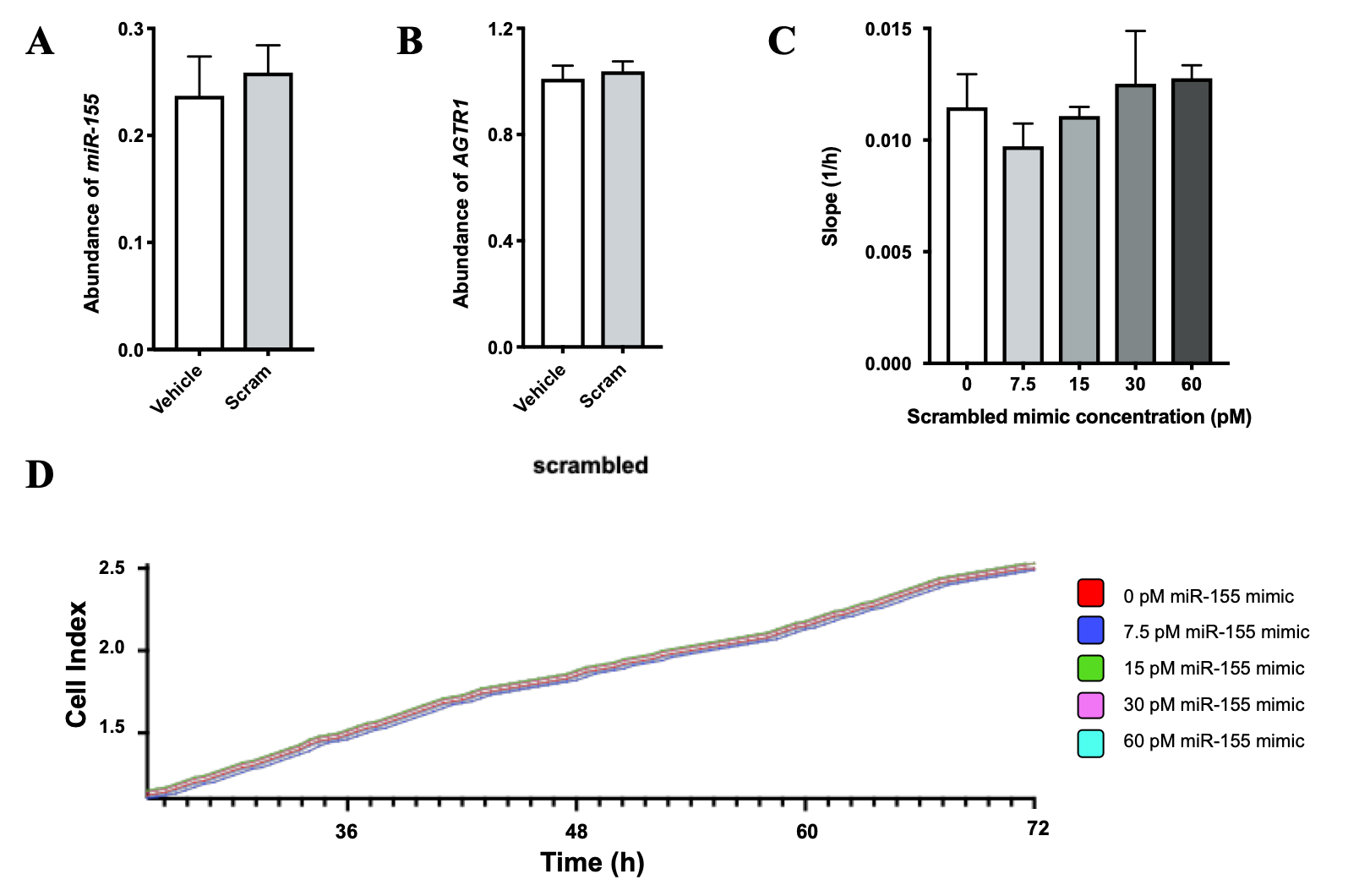
**

***Supplementary Figure 1.*** *The abundance of miR-155 and AGTR1 mRNA and cellular proliferation in HTR-8/SVneo cells treated with vehicle or scrambled miR-mimic.*

**A** *miR-155* expression and **B** *AGTR1* mRNA HTR-8/SVneo cells treated with vehicle or scrambled miR-mimic controls. **C** The rate of proliferation of cells, and **D** the trajectories for all scrambled mimic concentrations. *Data are presented as mean ± SEM. n = 3 experiments, each in triplicate.*
