# Supplementary material for: miR-155 suppresses angiotensin II type 1 receptor synthesis during placental morphogenesis
Source: Cell Death Discov. 2025 Dec 24;12:49. doi: 10.1038/s41420-025-02892-0 (PMC12847812; doi:10.1038/s41420-025-02892-0)
Supplement: Supplementary file 2 — Supplementary Figure 2 [file 41420_2025_2892_MOESM2_ESM.docx]

**
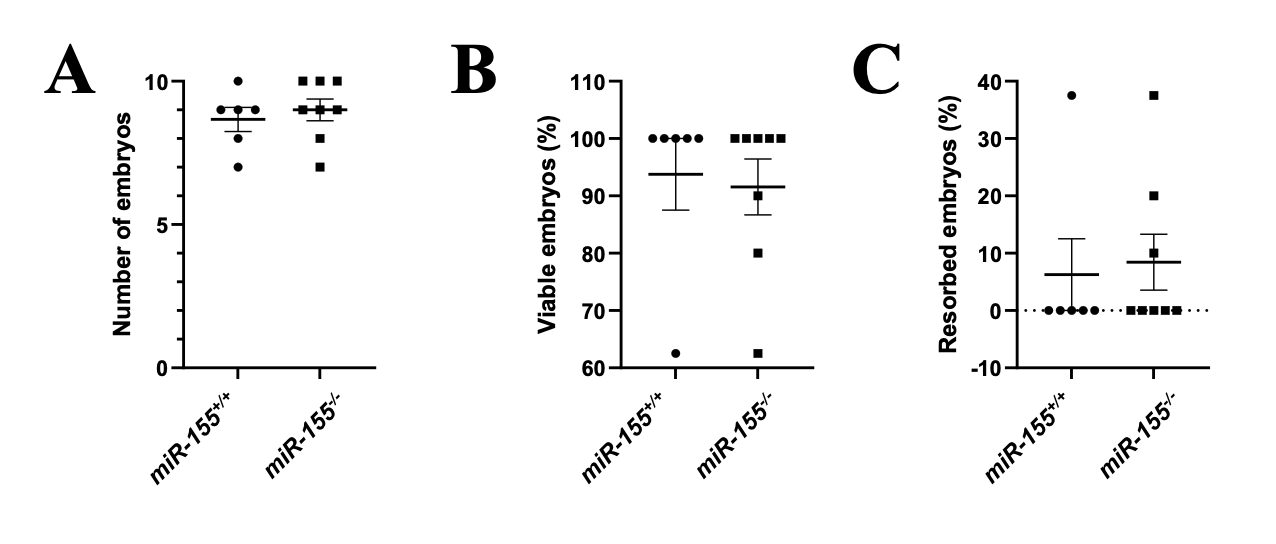
**

***Supplementary Figure 2.*** *Pregnancy viability in wild-type and miR-155^-/-^ dams.*

**A** Number of embryos per litter, **B** percentage of embryos that were viable, and **C** percentage of embryos that were resorbed. *Data are presented as scatter plots with mean ± SEM. n = 6 wild type dams, n= 8 miR-155^-/-^ dams.*
