# Supplementary material for: miR-155 suppresses angiotensin II type 1 receptor synthesis during placental morphogenesis
Source: Cell Death Discov. 2025 Dec 24;12:49. doi: 10.1038/s41420-025-02892-0 (PMC12847812; doi:10.1038/s41420-025-02892-0)
Supplement: Supplementary file 3 — Supplementary Figure 3 [file 41420_2025_2892_MOESM3_ESM.docx]

*
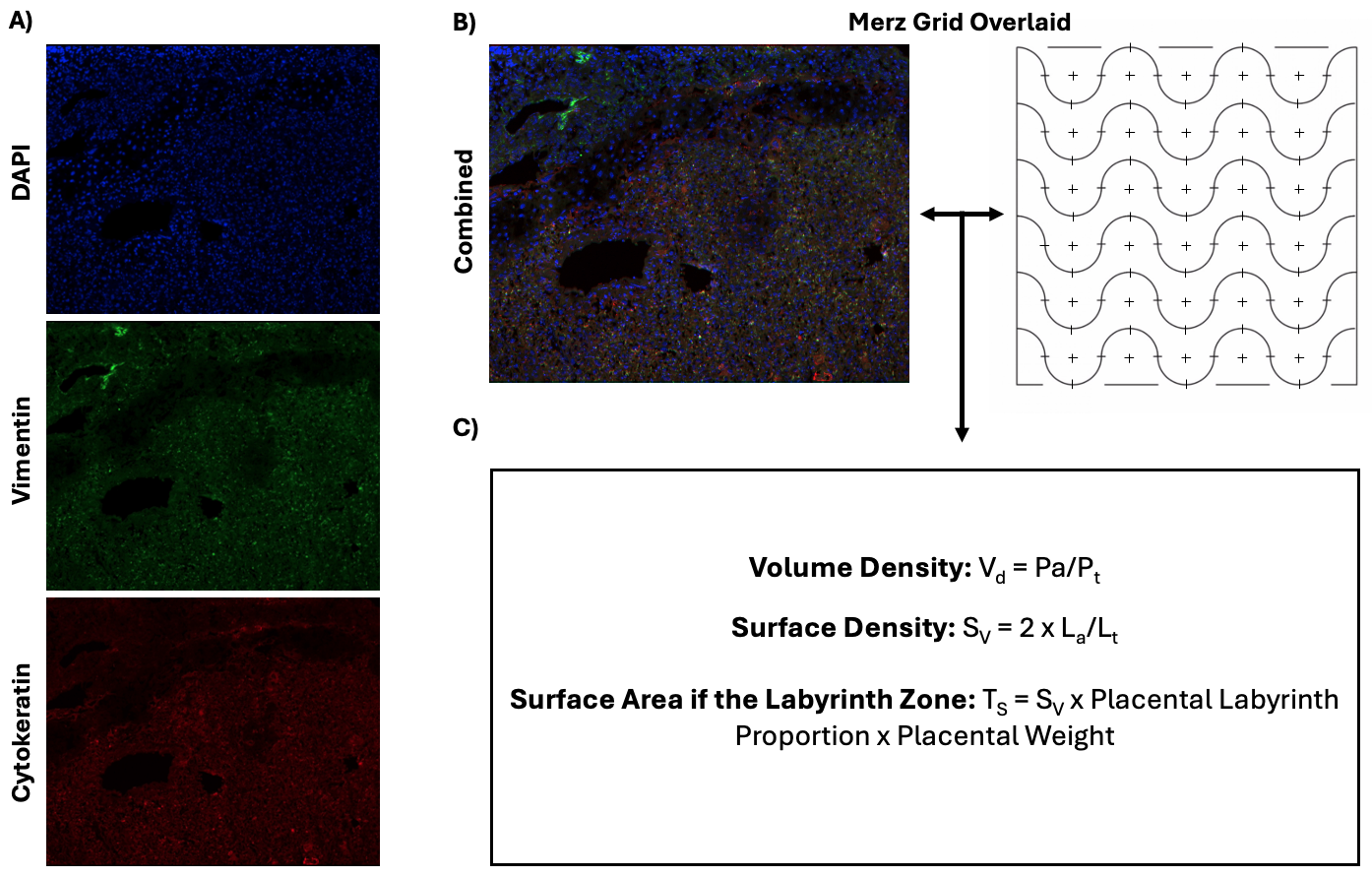
*

***Supplementary Figure 3.*** *Diagrammatic outline of stereological Merz grid analysis.*

**A** Mouse placental sections underwent immunofluorescent staining for the following: trophoblasts (Cytokeratin; Red), fetal capillaries (Vimentin; Green), and a nuclear DAPI stain (Blue). After staining, placental sections were assessed by immunofluorescent microscopy where images were acquired utilising uniform random sampling. **B** A stereological Merz Grid was applied to each image for analysis to determine volume density (V_d_), surface density (S_V_), and total surface area (T_S_) within the labyrinth of the components of interest (trophoblasts, fetal capillaries, and maternal blood space). All images were acquired at 20x magnification. Abbreviations: V_d_: Volume density. P_a_: total number of grid points per component. P_t_: total number of points applied to the image. S_V_: Surface density. L_a_: total number of line intercepts. L_t_: total length of line applied to the image. T_s_: Total surface area
