# Supplementary material for: miR-155 suppresses angiotensin II type 1 receptor synthesis during placental morphogenesis
Source: Cell Death Discov. 2025 Dec 24;12:49. doi: 10.1038/s41420-025-02892-0 (PMC12847812; doi:10.1038/s41420-025-02892-0)
Supplement: Supplementary file 5 — Supplementary Figure 5 [file 41420_2025_2892_MOESM5_ESM.docx]

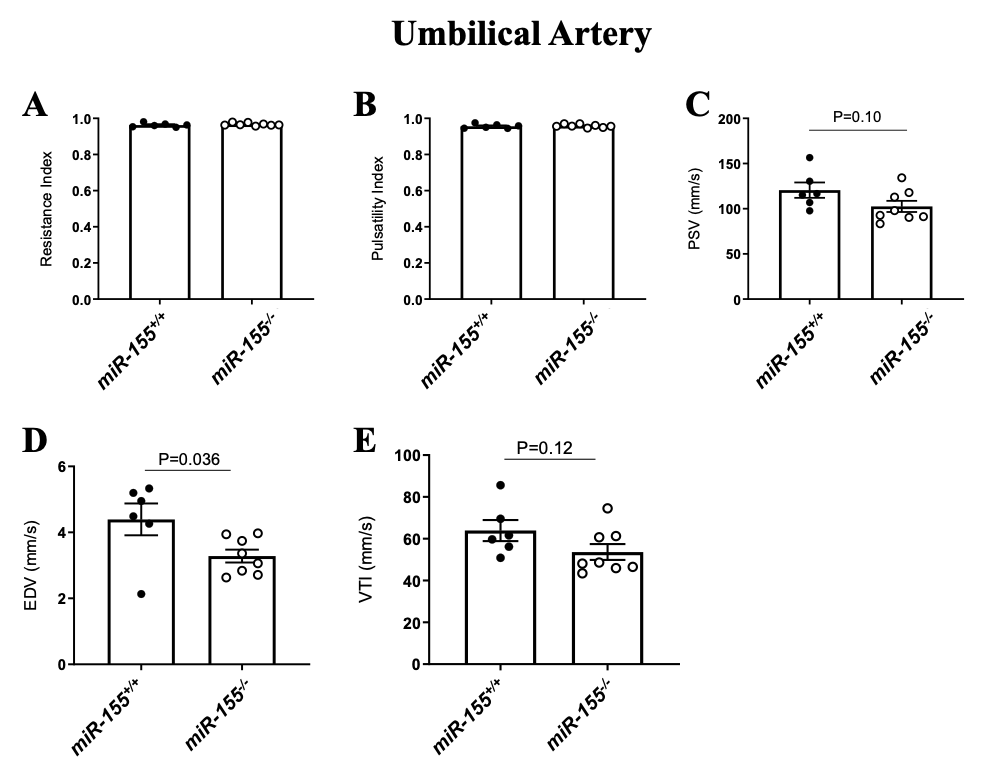


***Supplementary Figure 5.*** *Late-gestation umbilical artery Doppler parameters in miR-155^+/+^ and miR-155^-/-^ mice.*

Measurements were taken on pc day 17.5 from pregnant WT and *miR-155*^-/-^. **A** Resistance Index, and **B** Pulsatility Index, were calculated. **C** Peak-systolic velocity (PSV), **D** end-diastolic Velocity (EDV) and **E** velocity-time interval (VTI) were measured. *Data are presented as mean ± SEM. n = 6-8 dams/group and 3-4 umbilical arteries were measured per dam.*
