# Supplementary material for: miR-155 suppresses angiotensin II type 1 receptor synthesis during placental morphogenesis
Source: Cell Death Discov. 2025 Dec 24;12:49. doi: 10.1038/s41420-025-02892-0 (PMC12847812; doi:10.1038/s41420-025-02892-0)
Supplement: Supplementary file 6 — Supplementary Figure 6 [file 41420_2025_2892_MOESM6_ESM.docx]

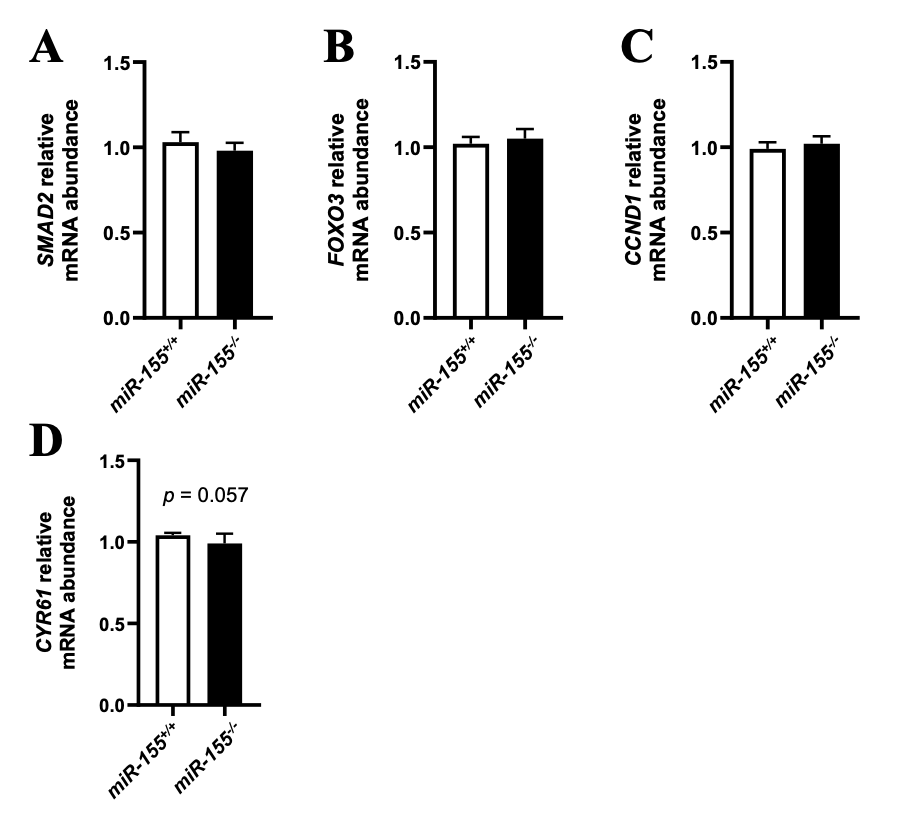


***Supplementary Figure 6.*** *The relative abundance of SMAD2, FOXO3, CCND1 and CYR61 mRNA in placentae from miR-155^+/+^ and miR-155^-/-^ mice.*

Relative abundance of **A** *SMAD2,* **B** *FOXO3,* **C** *CCND1* and **D** *CYR61* mRNA. *Data are presented as mean ± SEM. n = 3 experiments, each in triplicate.*
