# Supplementary material for: miR-155 suppresses angiotensin II type 1 receptor synthesis during placental morphogenesis
Source: Cell Death Discov. 2025 Dec 24;12:49. doi: 10.1038/s41420-025-02892-0 (PMC12847812; doi:10.1038/s41420-025-02892-0)
Supplement: Supplementary file 7 — Supplementary Figure 7 [file 41420_2025_2892_MOESM7_ESM.docx]

1. **
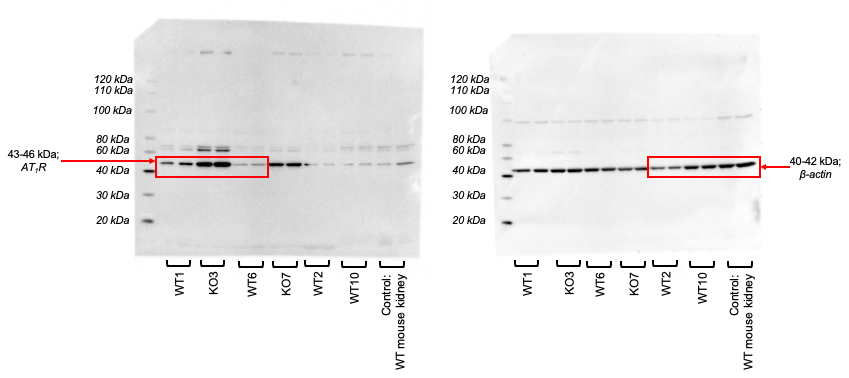
**

**B)**
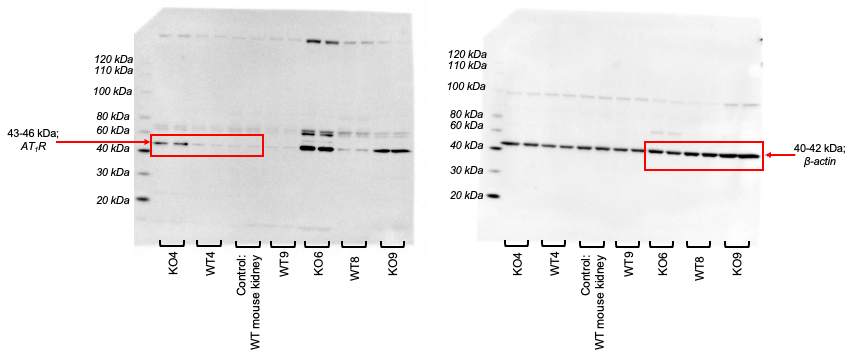


**C)
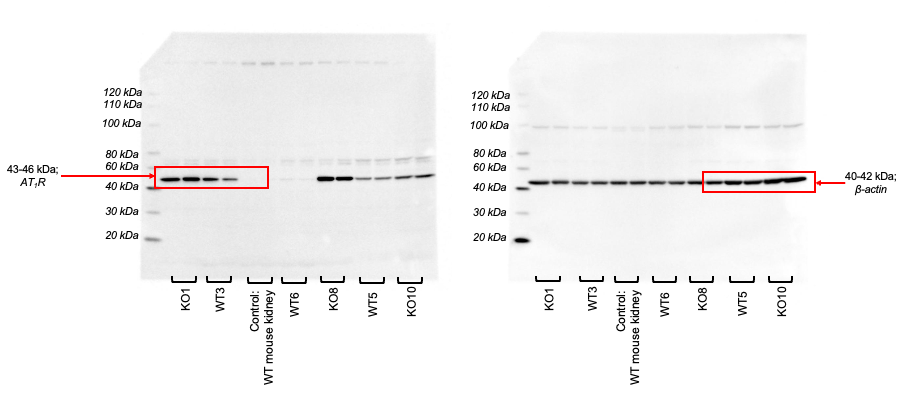
**

**D)
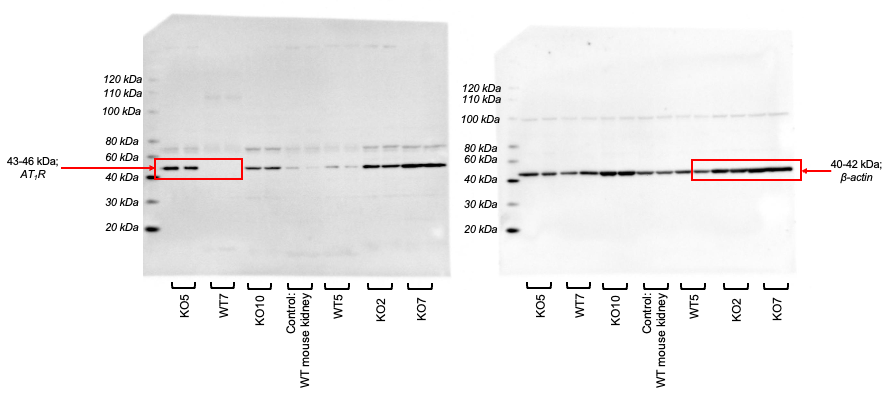
**

*Supplementary Figure 7: Representative full-length immunoblot images of AT_1_R and β-actin densitometry for mouse placentae.* AT_1_R was detected as a clear singular band between 43-46 kDa and β-actin was detected as a clear singular band at ~42 kDa. **A/B/C/D),** are full length blots of representative westerns. The red boxes depict bands shown in the representative images in those figures.
